# Supplementary material for: The Gut Bacteria Dysbiosis Contributes to Chronic Graft-Versus-Host Disease Associated With a Treg/Th1 Ratio Imbalance
Source: Front Microbiol. 2022 Sep 8;13:813576. doi: 10.3389/fmicb.2022.813576 (PMC9493085; doi:10.3389/fmicb.2022.813576)
Supplement: Supplementary file 1 [file Data_Sheet_1.zip › P101SC18090073-01-B1-3-4_result/05.WebShow/taxa_charts_html/charts/2xU0iOXneoOUlrILTxtrwN5yzrq5bH_legend.pdf]

k__Bacteria;Other;Other;Other;Other
k__Bacteria;p__Bacteroidetes;c__Bacteroidia;o__Bacteroidales;f__Bacteroidaceae
k__Bacteria;p__Bacteroidetes;c__Bacteroidia;o__Bacteroidales;f__Marinifilaceae
k__Bacteria;p__Bacteroidetes;c__Bacteroidia;o__Bacteroidales;f__Muribaculaceae
k__Bacteria;p__Bacteroidetes;c__Bacteroidia;o__Bacteroidales;f__Prevotellaceae
k__Bacteria;p__Bacteroidetes;c__Bacteroidia;o__Bacteroidales;f__Rikenellaceae
k__Bacteria;p__Bacteroidetes;c__Bacteroidia;o__Bacteroidales;f__Tannerellaceae
k__Bacteria;p__Firmicutes;c__Bacilli;o__Lactobacillales;f__Enterococcaceae
k__Bacteria;p__Firmicutes;c__Bacilli;o__Lactobacillales;f__Lactobacillaceae
k__Bacteria;p__Firmicutes;c__Clostridia;o__Clostridiales;Other
k__Bacteria;p__Firmicutes;c__Clostridia;o__Clostridiales;f__Lachnospiraceae
k__Bacteria;p__Firmicutes;c__Clostridia;o__Clostridiales;f__Peptococcaceae
k__Bacteria;p__Firmicutes;c__Clostridia;o__Clostridiales;f__Ruminococcaceae
k__Bacteria;p__Firmicutes;c__Clostridia;o__Clostridiales;f__unidentified_Clostridiales
k__Bacteria;p__Firmicutes;c__Erysipelotrichia;o__Erysipelotrichales;f__Erysipelotrichaceae
k__Bacteria;p__Proteobacteria;c__Deltaproteobacteria;o__Desulfovibrionales;f__Desulfovibrionaceae
k__Bacteria;p__Proteobacteria;c__Gammaproteobacteria;o__Enterobacteriales;f__Enterobacteriaceae
k__Bacteria;p__Tenericutes;c__Mollicutes;o__;f__
k__Bacteria;p__Verrucomicrobia;c__Verrucomicrobiae;o__Verrucomicrobiales;f__Akkermansiaceae
All Other Categories
